# *ABCG2* and *ABCC2* Expression Levels and Their Relationship With Disease Severity in Acute Pancreatitis

**DOI:** 10.1111/jcmm.71368

**Published:** 2026-09-16

**Authors:** Süleyman Baş, Esra Güzel Tanoğlu, Alpaslan Tanoğlu, Hafize Tuğba Karahan, Muhammed Said Gökçe, Murat Yeniçeri, Mustafa Can Şenoymak, Kadem Arslan

**Affiliations:** ^1^ Department of Internal Medicine University of Health Sciences, Sancaktepe Sehit Prof. Dr. Ilhan Varank Training and Research Hospital İstanbul Turkey; ^2^ Department of Molecular Biology and Genetics University of Health Sciences Istanbul Turkey; ^3^ Division of Gastroenterology Department of Internal Medicine Bahçeşehir University Istanbul Turkey; ^4^ Department of Internal Medicine Kütahya Evliya Çelebi Training and Research Hospital Kütahya Turkey; ^5^ Department of Biotechnology Institute of Health Sciences, Bezmialem Vakıf University Istanbul Turkey; ^6^ Department of Rheumatology University of Health Sciences, Kartal Dr. Lutfi Kırdar City Hospital İstanbul Turkey; ^7^ Department of Endocrinology and Metabolism University of Health Sciences, Sultan 2. Abdulhamid Han Training and Research Hospital İstanbul Turkey

**Keywords:** *ABCG2*, acute pancreatitis, ATP‐binding cassette transporters, computed tomography, disease severity, gene expression

## Abstract

The aim of this study was to evaluate the expression levels of *ABCG2* and *ABCC2*, members of the ATP‐binding cassette (ABC) transporter gene family, in patients with acute pancreatitis and to investigate their potential associations with disease presence as well as clinical and radiological disease severity. This prospective study included 80 patients diagnosed with acute pancreatitis and 96 healthy controls. Disease severity in acute pancreatitis was assessed using the Ranson score and computed tomography (CT) findings. *ABCG2* and *ABCC2* gene expression levels were measured in peripheral blood samples using quantitative real‐time polymerase chain reaction (qRT‐PCR). Statistical analyses, including between‐group comparisons and evaluations of associations between gene expression and disease severity, were performed using SPSS version 25.0. *ABCG2* gene expression levels were significantly lower in patients with acute pancreatitis than in healthy controls (*p* = 0.025). However, *ABCG2* expression was significantly higher in moderate and severe acute pancreatitis cases based on CT findings compared with mild cases (*p* = 0.038). *ABCC2* gene expression showed no significant association with either the presence of acute pancreatitis or disease severity. In ROC analysis, the area under the curve (AUC) for *ABCG2* gene expression was statistically significant (AUC = 0.598, *p* = 0.025). This study suggests that *ABCG2* gene expression may be associated with the presence of acute pancreatitis and radiological disease severity. *ABCG2* may represent a dynamically regulated candidate biomarker related to inflammatory and cellular stress processes in acute pancreatitis. Further studies including larger patient cohorts and preferably multicenter designs are needed to validate these findings.

## 1 Introduction

Acute pancreatitis is one of the most common gastrointestinal disorders requiring hospitalization worldwide, and its incidence is steadily increasing [1]. Although the vast majority of cases are mild, approximately 80% of patients recover without complications, whereas 20% develop serious local and systemic complications. This significantly increases morbidity and mortality rates [2].

In cases of acute pancreatitis, the process begins with abnormal enzyme activation in pancreatic acinar cells, leading to local inflammation. In more severe instances, this can progress to a systemic inflammatory response [2, 3, 4, 5, 6]. The pathogenesis of the disease involves key mechanisms such as the release of inflammatory cytokines, disruption of cellular calcium homeostasis, mitochondrial dysfunction and oxidative stress [7, 8, 9]. This heterogeneous clinical course makes predicting the prognosis in the early stages of the disease particularly challenging [2, 3].

Currently, the diagnosis of acute pancreatitis and the assessment of disease severity rely on a combined approach that includes clinical findings, serum biochemical parameters, clinical scoring systems and imaging‐based methods [1, 2, 3, 10]. However, each of these approaches has inherent limitations, and no single laboratory marker has yet been identified that can reliably reflect disease severity and accurately predict the subsequent clinical course on its own. Therefore, there remains a clear need to investigate novel molecular biomarkers that may better capture the underlying biological processes of acute pancreatitis [2].

ATP‐binding cassette (ABC) transporters constitute a large protein superfamily involved in maintaining cellular barrier functions and mediating the transmembrane transport of a wide range of endogenous and exogenous compounds. These transporters act as ATP‐dependent primary active pumps and play an important role in preserving cellular homeostasis and regulating cellular stress responses [11, 12]. Notably, the expression of ABC transporters has been shown to be modulated by inflammatory signalling and various cellular stress conditions [12, 13].

Among the subgroups of the ABC transporter family, the *ABCG* subfamily includes the *ABCG2* gene, which is located on chromosome 4q22 and mediates the efflux of various substrates out of the cell through an ATP‐dependent transport mechanism [11, 12, 13]. Another key member of this family, *ABCC2* (MRP2), is expressed not only in the liver but also in human pancreatic tissue, particularly on the apical membrane of ductal cells, where it contributes to the export of toxic metabolites and inflammatory mediators [14, 15]. Given these functional characteristics, both *ABCG2* and *ABCC2* are considered to be potentially linked to inflammatory responses and cellular stress pathways. However, the expression levels of ABC transporter genes in acute pancreatitis and their associations with clinical and radiological disease severity have not yet been sufficiently clarified. Therefore, the aim of the present study was to evaluate the expression levels of *ABCG2* and *ABCC2* in patients with acute pancreatitis and to investigate their potential relationship with disease presence as well as clinical and radiological severity.

## 2 Materials and Methods

### 2.1 Study Design and Participants

This study was designed as a prospective clinical study and was conducted at the Department of Internal Medicine, University of Health Sciences Sancaktepe Şehit Prof. Dr. İlhan Varank Training and Research Hospital. Patients aged 18–75 years who were hospitalized with a diagnosis of acute pancreatitis between December 2023 and January 2025 were prospectively enrolled. A total of 80 patients with acute pancreatitis and 96 healthy controls were included in the study. The control group consisted of healthy volunteers who were comparable to the patient group in terms of age and sex and had no known acute or chronic inflammatory disease, no acute or chronic infection, no rheumatologic disease and no chronic liver or kidney disease.

### 2.2 Diagnosis and Clinical Assessment of Acute Pancreatitis

The diagnosis of acute pancreatitis is established when at least two of the following three criteria are met: the presence of typical abdominal pain consistent with acute pancreatitis; serum amylase and/or lipase levels at least three times the upper limit of normal; and/or imaging findings compatible with the condition [16].

Clinical and laboratory evaluations of the patients with acute pancreatitis were performed routinely upon admission and at the 48th hour. For assessing clinical disease severity, the Ranson score was utilized [17].

### 2.3 Radiological Assessment

For the evaluation of radiological disease severity, computed tomography (CT) images obtained at the time of admission were reviewed to identify early structural alterations. However, to account for the fact that local complications and necrosis may undergo dynamic evolution during the early phase, the final severity classification was performed in strict accordance with the revised Atlanta classification by integrating both clinical and imaging follow‐up data. Specifically, mild AP was defined as the absence of organ failure and local/systemic complications. Moderately severe AP was defined by the presence of transient organ failure (resolving within 48 h) and/or local complications, whereas severe AP was characterized by persistent organ failure (> 48 h) evaluated via the modified Marshall scoring system. For patients displaying persistent clinical derangement or worsening inflammatory markers past 48–72 h, follow‐up CT imaging was performed and utilized to finalize the local complication status [16].

### 2.4 Sample Collection and Storage

Peripheral venous blood samples were obtained from patients and healthy controls during routine clinical evaluation. Following completion of clinical biochemical analyses, the remaining blood samples were centrifuged, separated, and stored at −80°C until molecular analyses were performed.

### 2.5 RNA Isolation

Total RNA was extracted from whole‐blood samples using the One Step‐RNA Reagent (Biobasic, Canada), following the manufacturer's instructions. Briefly, samples were homogenized in TRIzol, phase separation was achieved with chloroform, and RNA was precipitated from the aqueous phase using isopropanol. The resulting RNA pellets were washed with 75% ethanol and then dissolved in nuclease‐free water. RNA concentration and purity were assessed spectrophotometrically using a NanoDrop ND‐2000c instrument (Thermo Fisher Scientific Inc., Wilmington, DE).

### 2.6 cDNA Synthesis and Quantitative Real‐Time PCR

Complementary DNA (cDNA) was synthesized from equal amounts of total RNA using the OneScript Plus cDNA Synthesis Kit (ABM, Canada), according to the manufacturer's recommendations. The expression levels of *ABCG2* and *ABCC2* were determined using quantitative real‐time polymerase chain reaction (qRT‐PCR) with the BlasTaq 2X qPCR Master Mix (ABM, Canada). The β‐actin gene was used as an internal control. All PCR reactions were performed in triplicate, and relative gene expression levels were calculated using the 2^−^ΔΔCt method.

PCR cycling conditions consisted of an initial enzyme activation step at 95°C for 10 min, followed by 40 cycles of denaturation at 95°C for 15 s and annealing/extension at 60°C for 1 min.

### 2.7 Ethical Approval

The study protocol was approved by the Local Ethics Committee of the University of Health Sciences Sancaktepe Şehit Prof. Dr. İlhan Varank Training and Research Hospital (Date: 20 September 2023; Approval No: 2023/168). Written informed consent was obtained from all participants, and the study was conducted in accordance with the principles of the Declaration of Helsinki.

### 2.8 Statistical Analysis

Statistical analyses were performed using SPSS for Windows, version 25.0 (IBM Corp., Armonk, NY, USA). The distribution of continuous variables was assessed using the Kolmogorov–Smirnov and Shapiro–Wilk tests. Continuous variables with a normal distribution were presented as mean ± standard deviation, whereas non‐normally distributed variables were expressed as median [interquartile range]. Comparisons of continuous variables between two independent groups were carried out using the independent samples *t*‐test for normally distributed data and the Mann–Whitney *U* test for non‐normally distributed data. Categorical variables were compared using the chi‐square test.

Receiver operating characteristic (ROC) curve analysis was performed to evaluate the discriminatory performance of *ABCG2* gene expression levels for acute pancreatitis, and the area under the curve (AUC) was calculated. In the ROC analysis, the positive state was defined such that lower values of the test variable indicated positivity. A *p*‐value < 0.05 was considered statistically significant.

## 3 Results

A total of 80 acute pancreatitis patients and 96 healthy controls were included in the study. The mean age of the acute pancreatitis group was 49.74 ± 15.90 years, whereas that of the control group was 47.96 ± 14.90 years. No statistically significant difference was found between the two groups regarding age (*p* = 0.445). In the acute pancreatitis group, 60% of the patients were male and 40% were female; in the control group, these rates were 62.5% and 37.5%, respectively. No significant difference was observed between the groups in terms of gender distribution (*p* = 0.734). Regarding the etiology of acute pancreatitis, gallstones were the most common cause (52.5%), followed by alcohol use (15%), hyperlipidemia (5%), medication‐related causes (3.75%) and other etiologies (23.75%) (Table 1).

**TABLE 1:** Demographic characteristics and etiological factors of the acute pancreatitis and control groups.

	Acute pancreatitis group (*n* = 80)	Control group (*n* = 96)	*p*
Age (year)	49.74 ± 15.90^a^	47.96 ± 14.90^a^	0.445^b^
Gender (M/F)	48 (60%)/32 (40%)	60 (62.5%)/36 (37.5%)	0.734^c^
Etiological factors			
Biliary	42 (52.5%)	NA	NA
Alcohol	12 (15%)	NA	NA
Hyperlipidemia	4 (5%)	NA	NA
Drugs	3 (3.75%)	NA	NA
Other	19 (23.75%)	NA	NA

Abbreviation: NA, not applicable in the control group/no comparison performed.

^a^ Mean ± standard deviation.

^b^ Independent *t*‐test.

^c^ Chi‐square test.

In patients with acute pancreatitis, clinical severity assessment showed a median Ranson score of 1 [0–2] at admission, 2 [1–3] at 48 h, and a median total Ranson score of 3 [1–5]. Based on the Ranson score, 57.5% (*n* = 46) of patients were classified as having severe acute pancreatitis (score ≥ 3). According to radiological severity classification, evaluation of computed tomography findings indicated that 83.7% (*n* = 67) of patients had mild acute pancreatitis, whereas 16.3% (*n* = 13) had moderate or severe acute pancreatitis. When clinical outcomes and complications were examined, pleural effusion was observed in 11.3% (*n* = 9) of patients. The need for supplemental oxygen was noted in 7.5% (*n* = 6), and intensive care unit admission was required in 5% (*n* = 4) of cases. The median length of hospital stay was 6 [4–11.75] days (Table 2).

**TABLE 2:** Disease severity, radiological classification and clinical outcomes in patients with acute pancreatitis.

	Acute pancreatitis (*n* = 80)
Clinical severity assessment	
Ranson score (at admission)	1 [0–2]^a^
Ranson score (at 48th hour)	2 [1–3]^a^
Ranson score (total)	3 [1–5]^a^
Severe AP according to Ranson score (score ≥ 3)	46 (57.5%)
Radiological severity classification	
Mild acute pancreatitis according to CT	67 (83.7%)
Moderate and severe acute pancreatitis according to CT	13 (16.3%)
Clinical outcomes and complications	
Pleural effusion	9 (11.3%)
Supplemantal oxygen requirement	6 (7.5%)
ICU requirement	4 (5%)
Length of hospitalization (days)	6 [4–11.75]^a^

Abbreviations: AP, acute pancreatitis; CT, computed tomography; ICU, intensive care unit.

^a^ Median [interquartile range (IQR)].

*ABCG2* and *ABCC2* gene expression levels were compared between patients with acute pancreatitis and healthy controls. The median *ABCG2* expression level was 0.29 [0.08–1.36] in the acute pancreatitis group, compared with 0.58 [0.16–4.28] in the control group. *ABCG2* expression differed significantly between the two groups (*p* = 0.025). In contrast, the median *ABCC2* expression level was 1.54 [0.09–58.28] in the acute pancreatitis group and 0.89 [0.07–15.50] in the control group, and no statistically significant difference was observed between groups (*p* = 0.172) (Table 3).

**TABLE 3:** Comparison of *ABCG2* and *ABCC2* gene expression levels between patients with acute pancreatitis and the control group.

	Acute pancreatitis group (*n* = 80)	Control group (*n* = 96)	*p*
*ABCG2*	0.29 [0.08–1.36]^a^	0.58 [0.16–4.28]^a^	**0.025** ^b^
*ABCC2*	1.54 [0.09–58.28]^a^	0.89 [0.07–15.50]^a^	0.172^b^

*Note:* Bold values are statistically significant (*p* < 0.05).

^a^ Median [interquartile range (IQR)].

^b^ Mann–Whitney *U* test.

In patients with acute pancreatitis, the relationship between disease severity and *ABCG2* and *ABCC2* gene expression was evaluated based on the Ranson score and CT findings. According to the Ranson score, no statistically significant difference in *ABCG2* expression levels was observed between the mild and severe acute pancreatitis groups (*p* = 0.098). Similarly, *ABCC2* expression levels did not differ significantly between groups stratified by the Ranson score (*p* = 0.392) (Table 4). In contrast, when disease severity was assessed according to CT findings, *ABCG2* expression levels were significantly higher in the moderate and severe acute pancreatitis group compared with the mild acute pancreatitis group (*p* = 0.038). *ABCC2* expression levels, however, showed no significant difference between CT‐based severity groups (*p* = 0.819) (Table 5).

**TABLE 4:** ABCG2 and ABCC2 gene expression levels according to disease severity based on the Ranson score.

	Mild AP according to Ranson score (*n* = 34)	Severe AP according to Ranson score (*n* = 46)	*p*
*ABCG2*	0.20 [0.05–0.97]^a^	0.44 [0.11–2.56]^a^	0.098^b^
*ABCC2*	2.61 [0.11–2838.38]^a^	1.21 [0.09–13.25]^a^	0.392^b^

Abbreviation: AP, acute pancreatitis.

^a^ Median [interquartile range (IQR)].

^b^ Mann–Whitney *U* test.

**TABLE 5:** *ABCG2* and *ABCC2* gene expression levels according to CT‐based disease severity.

	Mild AP according to CT (*n* = 67)	Moderate and severe AP according to CT (*n* = 13)	*p*
*ABCG2*	0.21 [0.07–1.13]^a^	1.05 [0.20–4.66]^a^	**0.038** ^b^
*ABCC2*	1.41 [0.08–154.52]^a^	2.06 [0.42–12.83]^a^	0.819^b^

*Note:* Bold values are statistically significant (*p* < 0.05).

Abbreviations: AP, acute pancreatitis; CT, computed tomography.

^a^ Median [interquartile range (IQR)].

^b^ Mann–Whitney *U* test.

**FIGURE 1:**
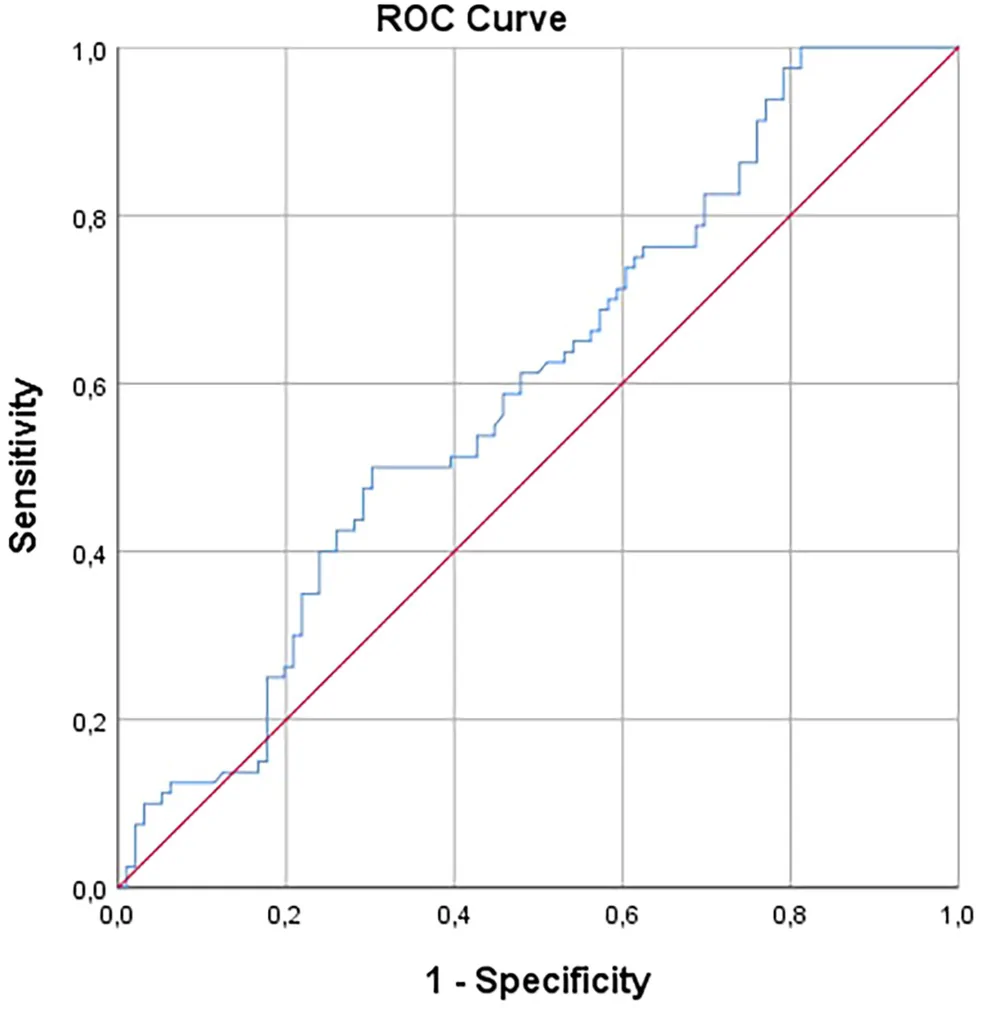
ROC curve showing the discriminative performance of *ABCG2* gene expression levels for acute pancreatitis.

The discriminatory performance of *ABCG2* gene expression levels in patients with acute pancreatitis was evaluated using ROC curve analysis. The AUC for *ABCG2* was 0.598 (95% CI: 0.515–0.682). This AUC value was found to be statistically significant (*p* = 0.025) (Table 6, Figure 1).

**TABLE 6:** ROC analysis of *ABCG2* gene expression levels in patients with acute pancreatitis.

	AUC (95% CI)	Standard error	*p*
*ABCG2*	0.598 (0.515–0.682)	0.043	**0.025**

*Note:* Bold values are statistically significant (*p* < 0.05).

Abbreviations: AUC, area under the curve; CI, confidence interval.

## 4 Discussion

To the best of our knowledge, this is the first study which evaluates *ABCG2* and *ABCC2* expression levels in acute pancreatitis patients. In this study, we evaluated the expression levels of *ABCG2* and *ABCC2* in patients with acute pancreatitis and explored their potential associations with disease presence as well as clinical and radiological severity. Our findings indicate that *ABCG2* expression was significantly lower in patients with acute pancreatitis compared with healthy controls. Interestingly, however, *ABCG2* expression was significantly higher in moderate and severe cases than in mild cases when severity was classified based on computed tomography findings. In contrast, *ABCC2* expression did not show a significant difference either in terms of disease presence or severity, suggesting that *ABCC2* may play a more limited role in the pathophysiology of acute pancreatitis.

Clinical scoring systems and imaging‐based classifications are widely used to establish the diagnosis and assess the severity of acute pancreatitis. In particular, computed tomography is considered a key tool for evaluating pancreatic necrosis and local complications [16, 18]. In the present study, we examined gene expression findings alongside both clinical and radiological severity assessments, aiming to provide a more comprehensive evaluation of the potential relationship between ABC transporter genes and disease severity in acute pancreatitis.

ABC transporters represent a large protein family involved in cellular barrier functions and in the transport of a wide range of endogenous and exogenous compounds across cell membranes [19]. Their expression has been shown to be influenced by cellular stress conditions, inflammatory responses and various environmental stimuli [20, 21]. In acute pancreatitis, early pathogenic events such as the inflammatory response, disruption of intracellular calcium homeostasis, mitochondrial dysfunction and oxidative stress play a central role [3]. In this context, it is plausible that the inflammatory and cellular stress responses occurring during acute pancreatitis may also affect the expression of ABC transporter genes.

Evidence on ABC transporter genes in pancreatitis remains scarce, and most available data come from experimental animal models. In a rat model of chronic pancreatitis, Güzel Tanoğlu et al. reported that *ABCG2* and *ABCC2* expression levels changed in the presence of chronic inflammation, and that melatonin administration influenced oxidative stress and endoplasmic reticulum stress parameters associated with the expression of these genes [22]. In contrast, in the present study using samples from patients with acute pancreatitis, *ABCG2* expression was suppressed compared with the control group, yet expression levels increased as disease severity worsened according to CT findings. These differences may be explained by distinct biological processes underlying acute versus chronic pancreatitis, interspecies variation (human vs. animal), and differences in sampling methods (peripheral blood vs. experimental pancreatic tissue). It is possible that the intense cytokine response and cellular stress during the acute inflammatory phase initially suppress *ABCG2* expression, whereas a compensatory upregulation may occur in more severe cases. Overall, these findings suggest that *ABCG2* may represent a molecular marker that is dynamically regulated throughout the inflammatory and cellular stress response in acute pancreatitis [20, 21].

In a comprehensive review, Criddle emphasized that an increase in reactive oxygen species (ROS), together with disruption of intracellular Ca^2+^ homeostasis, leads to mitochondrial dysfunction, ATP depletion and ultimately acinar cell necrosis [23]. These processes may also influence the expression of the ABC transporter family, which plays a central role in cellular detoxification and stress responses. In our study, the finding that *ABCG2* expression was suppressed in patients with acute pancreatitis compared with healthy controls appears consistent with an impaired adaptive response during the early phase of oxidative and mitochondrial stress described by Criddle. On the other hand, the observation that *ABCG2* expression levels were higher in moderate and severe acute pancreatitis than in mild cases based on CT findings may reflect a late compensatory response to the increasing oxidative burden and more pronounced cellular injury in advanced stages of the disease. In experimental models, Li et al. [24] demonstrated a protective role for *ABCG2*, showing that it can reduce oxidative stress and apoptosis. Although their study focused on epilepsy and the neurological system, the molecular regulatory pathways linking *ABCG2* to oxidative stress may still be relevant to our results. Overall, our findings suggest that *ABCG2* may be involved in the cellular stress response in acute pancreatitis and could be related to disease severity.

In analyses where clinical severity was assessed using the Ranson score, *ABCG2* expression tended to be higher in the severe group but did not reach statistical significance, which may be related to the limited ability of clinical scoring systems to fully capture disease severity in the early phase [17]. In contrast, in the CT‐based radiological classification, *ABCG2* expression was significantly higher in the moderately severe/severe acute pancreatitis group. This may be explained by the fact that radiological assessment can more directly demonstrate morphological injury, including pancreatic necrosis and local complications [16, 18]. These findings suggest that *ABCG2* may show a stronger association with radiological severity indicators than with clinical scoring systems.

The ROC analysis yielded an AUC of 0.598 for *ABCG2* gene expression, indicating a statistically significant, although modest, discriminatory performance for distinguishing acute pancreatitis. However, the observation that *ABCG2* expression was lower in patients with acute pancreatitis compared with healthy controls suggests that this gene may be less useful as a stand‐alone diagnostic marker and may instead reflect the underlying inflammatory and cellular stress processes involved in acute pancreatitis. In this context, the ROC findings support the potential of *ABCG2* as a biomarker reflecting disease biology; however, its clinical utility should be confirmed in larger patient cohorts, preferably in multicenter studies.

Several limitations should be considered when interpreting our findings. The study was single‐centre with a limited sample size, and gene expression analyses were performed on peripheral blood samples instead of pancreatic tissue. Nevertheless, the study also has notable strengths. It is among the limited number of studies evaluating *ABCG2* and *ABCC2* gene expression in acute pancreatitis using human clinical samples. In addition, analysing gene expression findings together with clinical severity scores and radiological severity classification allowed us to link molecular data with clinical and imaging features, thereby strengthening the clinical relevance of our results. Overall, our study provides original data that may contribute to a better understanding of the molecular mechanisms involved in acute pancreatitis.

## 5 Conclusion

This study suggests that *ABCG2* gene expression may be associated with both the presence of acute pancreatitis and radiological disease severity. *ABCG2* expression was significantly lower in patients with acute pancreatitis than in healthy controls, whereas *ABCG2* levels were significantly higher in moderate and severe cases based on computed tomography findings. In contrast, *ABCC2* gene expression showed no significant association with either disease presence or disease severity. Overall, these findings indicate that *ABCG2* may represent a dynamically regulated candidate molecular biomarker related to inflammatory and cellular stress processes in acute pancreatitis. However, further studies including larger patient cohorts and preferably multicenter designs are needed to confirm these results and to better define the potential clinical and prognostic value of *ABCG2*.

## Author Contributions

**Süleyman Baş:** conceptualization, investigation, resources, data curation, writing – original draft, writing – review and editing. **Esra Güzel Tanoğlu:** methodology, data curation, writing – review and editing. **Alpaslan Tanoğlu:** conceptualization, methodology, investigation, writing – review and editing. **Hafize Tuğba Karahan:** investigation, writing – original draft. **Muhammed Said Gökçe:** methodology. **Murat Yeniçeri:** resources. **Mustafa Can Şenoymak:** writing – original draft. **Kadem Arslan:** writing – review and editing.

## Funding

The authors have nothing to report.

## Disclosure

Use of Artificial Intelligence for Generating Text: The authors declare that they have not used any type of generative artificial intelligence for the writing of this manuscript, nor for the creation of images, graphics, tables, or their corresponding captions.

## Ethics Statement

Protection of Human and Animal Subjects: The authors declare that the procedures followed were in accordance with the regulations of the relevant clinical research ethics committee and with those of the Code of Ethics of the World Medical Association (Declaration of Helsinki).

## Consent

Written informed consent was obtained from all participants. The Local Ethics Committee of Sancaktepe Sehit Prof. Dr. Ilhan Varank Training and Research Hospital (Decision No: 2023/168, Date: 20.09.2023) approved the study.

## Conflicts of Interest

The authors declare no conflicts of interest.

## Data Availability

The datasets generated and/or analysed during the current study are not publicly available due to privacy and ethical restrictions but are available from the corresponding author upon reasonable request.

## References

[1] M. Schiemer , M. Treiber , and S. Heeg , “Akute Pankreatitis [Acute Pancreatitis],” Deutsche Medizinische Wochenschrift 146, no. 4 (2021): 229–236. 33592658 10.1055/a-1221-7186

[2] L. Boxhoorn , R. P. Voermans , S. A. Bouwense , et al., “Acute Pancreatitis,” Lancet 396, no. 10252 (2020): 726–734. 32891214 10.1016/S0140-6736(20)31310-6

[3] Z. Zheng , Y. X. Ding , Y. X. Qu , F. Cao , and F. Li , “A Narrative Review of Acute Pancreatitis and Its Diagnosis, Pathogenetic Mechanism, and Management,” Annals of Translational Medicine 9, no. 1 (2021): 69. 33553362 10.21037/atm-20-4802 PMC7859757

[4] A. Tanoglu , Y. Yazgan , M. Kaplan , et al., “Trimetazidine Significantly Reduces Cerulein‐Induced Pancreatic Apoptosis,” Clinics and Research in Hepatology and Gastroenterology 39, no. 1 (2015): 145–150. 25001186 10.1016/j.clinre.2014.06.003

[5] H. T. Karahan , A. Tanoglu , E. G. Tanoglu , M. S. Gokce , and E. Karahan , “Evaluation of Metastasis‐Associated Lung Adenocarcinoma Transcript 1 (MALAT 1) and H19 in Determining the Diagnosis and Severity of the Disease in Patients With Acute Pancreatitis,” Turkish Journal of Gastroenterology 37, no. 3 (2025): 347–353. 10.5152/tjg.2025.24701 PMC12994419 41846475

[6] Z. Cirak , A. Tanoglu , M. Yeniceri , E. G. Tanoglu , M. Kaplan , and A. G. Sade , “Certolizumab Has Favorable Efficacy on Preventing Pancreas and Target Organs Damage in Acute Pancreatitis,” Pancreas 53, no. 7 (2024): e588–e594. 38986079 10.1097/MPA.0000000000002343

[7] M. Yeniçeri , A. Tanoğlu , M. Salmanoğlu , et al., “Efficacy of Agmatine Treatment in Experimental Acute Pancreatitis Rat Model,” Turkish Journal of Gastroenterology 35, no. 1 (2024): 27–31. 10.5152/tjg.2024.23017 PMC10837605 38454275

[8] B. Ç. Güney , A. Tanoğlu , M. Yeniçeri , et al., “Favorable Efficacy of Adalimumab Treatment in Experimental Acute Pancreatitis Model,” Turkish Journal of Medical Sciences 52, no. 6 (2022): 1821–1828. 36945982 10.55730/1300-0144.5528 PMC10390199

[9] M. Kaplan , A. Tanoğlu , B. Çakır Güney , et al., “Golimumab Ameliorates Pancreatic Inflammatory Response in the Cerulein‐Induced Acute Pancreatitis in Rats,” Turkish Journal of Gastroenterology 33, no. 11 (2022): 918–924. 10.5152/tjg.2022.21456 PMC9797786 36262104

[10] M. Kiyak and A. Tanoglu , “Comparison of the Efficacy of Balthazar Score and C‐Reactive Protein‐Albumin Ratio for Determination of Acute Pancreatitis Severity,” Current Health Sciences Journal 48, no. 1 (2022): 81–87. 35911938 10.12865/CHSJ.48.01.12 PMC9289588

[11] R. Allikmets , L. M. Schriml , A. Hutchinson , V. Romano‐Spica , and M. Dean , “A Human Placenta‐Specific ATP‐Binding Cassette Gene (ABCP) on Chromosome 4q22 That is Involved in Multidrug Resistance,” Cancer Research 58, no. 23 (1998): 5337–5339. 9850061

[12] A. A. Stavrovskaya and T. P. Stromskaya , “Transport Proteins of the ABC Family and Multidrug Resistance of Tumor Cells,” Biochemistry (Mosc) 73, no. 5 (2008): 592–604. 18605983 10.1134/s0006297908050118

[13] A. W. Ravna and G. Sager , “Molecular Modeling Studies of ABC Transporters Involved in Multidrug Resistance,” Mini Reviews in Medicinal Chemistry 9, no. 2 (2009): 186–193. 19200023 10.2174/138955709787316065

[14] A. T. Nies and D. Keppler , “The Apical Conjugate Efflux Pump ABCC2 (MRP2),” Pflügers Archiv 453, no. 5 (2007): 643–659. 16847695 10.1007/s00424-006-0109-y

[15] J. König , M. Hartel , A. T. Nies , et al., “Expression and Localization of Human Multidrug Resistance Protein (ABCC) Family Members in Pancreatic Carcinoma,” International Journal of Cancer 115, no. 3 (2005): 359–367. 15688370 10.1002/ijc.20831

[16] P. A. Banks , T. L. Bollen , C. Dervenis , et al., “Classification of Acute Pancreatitis—2012: Revision of the Atlanta Classification and Definitions by International Consensus,” Gut 62, no. 1 (2013): 102–111. 23100216 10.1136/gutjnl-2012-302779

[17] J. H. Ranson , K. M. Rifkind , D. F. Roses , S. D. Fink , K. Eng , and F. C. Spencer , “Prognostic Signs and the Role of Operative Management in Acute Pancreatitis,” Surgery, Gynecology & Obstetrics 139, no. 1 (1974): 69–81. 4834279

[18] E. J. Balthazar , D. L. Robinson , A. J. Megibow , and J. H. Ranson , “Acute Pancreatitis: Value of CT in Establishing Prognosis,” Radiology 174, no. 2 (1990): 331–336. 2296641 10.1148/radiology.174.2.2296641

[19] M. Dean , Y. Hamon , and G. Chimini , “The Human ATP‐Binding Cassette (ABC) Transporter Superfamily,” Journal of Lipid Research 42, no. 7 (2001): 1007–1017. 11441126

[20] S. Kukal , D. Guin , C. Rawat , et al., “Multidrug Efflux Transporter ABCG2: Expression and Regulation,” Cellular and Molecular Life Sciences 78, no. 21–22 (2021): 6887–6939. 34586444 10.1007/s00018-021-03901-y PMC11072723

[21] G. K. Grewal , S. Kukal , N. Kanojia , L. Saso , S. Kukreti , and R. Kukreti , “Effect of Oxidative Stress on ABC Transporters: Contribution to Epilepsy Pharmacoresistance,” Molecules 22, no. 3 (2017): 365. 28264441 10.3390/molecules22030365 PMC6155434

[22] E. Güzel Tanoğlu , A. Tanoğlu , M. Ç. Meriçöz Aydın , and M. F. Esen , “Melatonin Has Favorable Preventive Effects on Experimental Chronic Pancreatitis Rat Model,” Turkish Journal of Medical Sciences 51, no. 5 (2021): 2734–2740. 34247466 10.3906/sag-2103-134 PMC8742498

[23] D. N. Criddle , “Reactive Oxygen Species, Ca(2+) Stores and Acute Pancreatitis; A Step Closer to Therapy?,” Cell Calcium 60, no. 3 (2016): 180–189. 27229361 10.1016/j.ceca.2016.04.007

[24] C. Li , Y. Cai , Y. Chen , et al., “ABCG2 Shields Against Epilepsy, Relieves Oxidative Stress and Apoptosis via Inhibiting the ISGylation of STAT1 and mTOR,” Redox Biology 75 (2024): 103262. 38981367 10.1016/j.redox.2024.103262 PMC11280404

